# Supplementary material for: Antibacterial and Antispore Activities of Isolated Compounds from Piper cubeba L
Source: Molecules. 2019 Aug 26;24(17):3095. doi: 10.3390/molecules24173095 (PMC6749514; doi:10.3390/molecules24173095)
Supplement: Supplementary file 1 [file molecules-24-03095-s001.pdf]

SUPPLEMENTARY MATERIALS

Table S1. Comparison of ^{1}H-NMR (500 MHz, CDCL$_3$) and ^{13}C-NMR (125 MHz, CDCL$_3$) of compound (**1**) with literature values.

Position	$^{\delta}$H	$^{\delta}$C	HMBC ^{2}J	HMBC ^{3}J	$^{\delta}$H* (500 MHz, CDCL$_3$	$^{\delta}$C* (125 MHz CDCL$_3$)
**1**	-	143.3				142.3
**2**	-	148.7				148.5
**3**	6.49 (1H, s, H-3)	97.8	C-2, C-4	C-1, C-5	6.49 (1H, dq, *J* = 11.4Hz and 1.83Hz, H-1’)	97.4
**4**	-	150.6			-	151.5
**5**	-	118.9			-	118.0
**6**	6.94 (1H, s, H-6)	114.6	C-1, C-5	C-7, C-2, C-4	6.84 (1H, s, H-6)	114.1
**7**	6.66 (1H, s, H-7)	125.0			6.53 (1H, s, H-3)	125.8
**8**	6.13 (1H, m, H-8)	124.4	C-7	C-5	5.76 (1H, dq, *J* = 12.0 Hz and 6.8Hz, H-2’)	124.7
**9**	1.89 (3H, dd, *J* = 8.0, 8.5Hz, H-9)	18.8	C-8		1.84 (3H, dd, *J* = 7.3Hz and 1.8Hz,	18.6
**10**	3.86 (3H, s, H-10)	56.4	C-1		3.83 (3H, s, OCH$_3$)	56.4
**11**	3.88 (3H, s, H-11)	56.1	C-2		3.89 (3H, s, OCH$_3$)	56.0
**12**	3.82 (3H, s, H-12)	56.7	C-4		3.81 (3H, s, OCH$_3$)	56.6

***McGaw *et al.* (2002) & Dung *et al* (2007)**

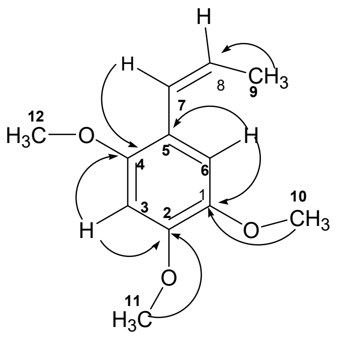

Figure S1. Structure and selected HMBC correlation of β-asarone isolated from *P. cubeba* L.

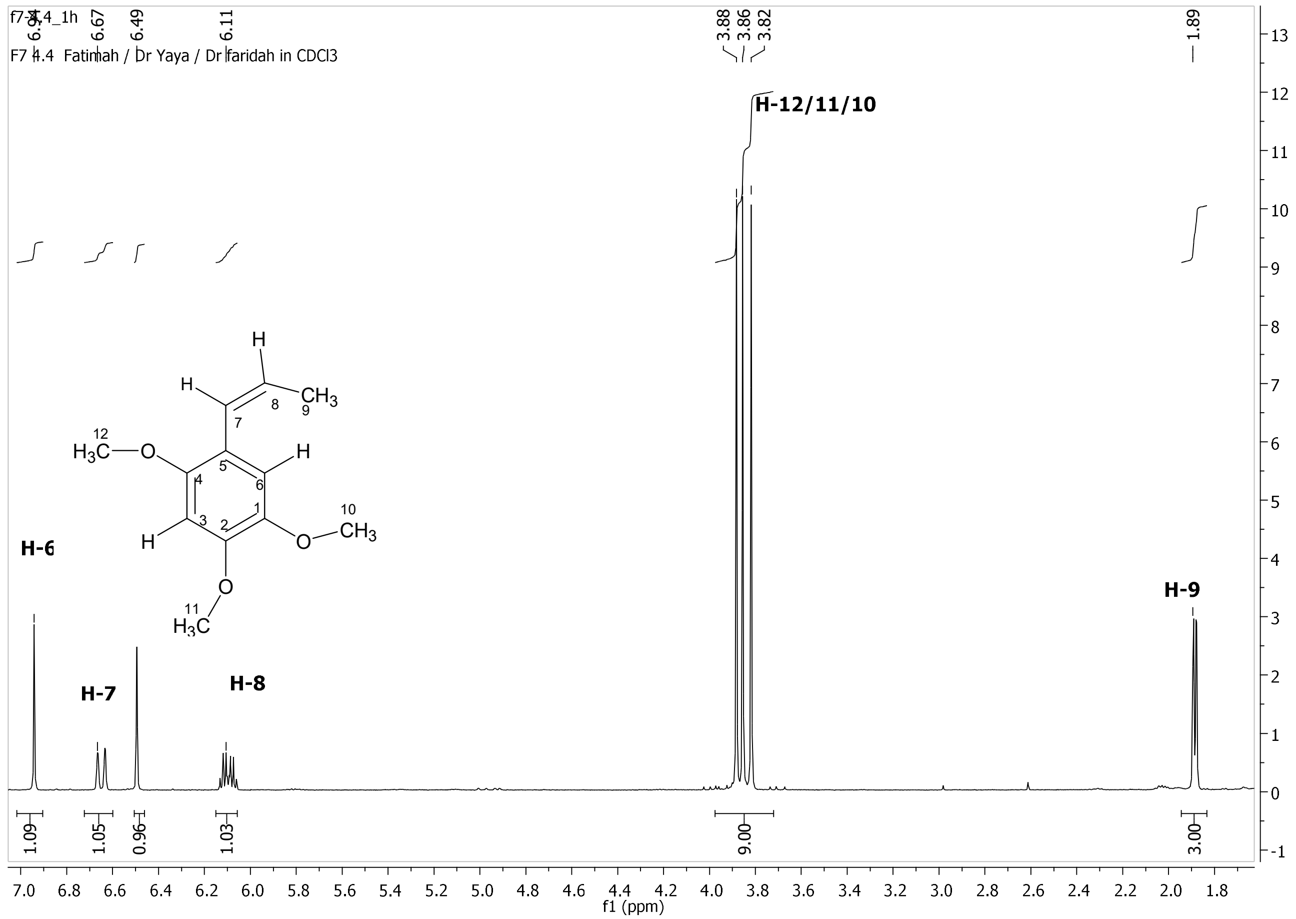

Figure S2. ^{1}H-NMR spectrum of β-asarone (500 MHz, CDCL3).

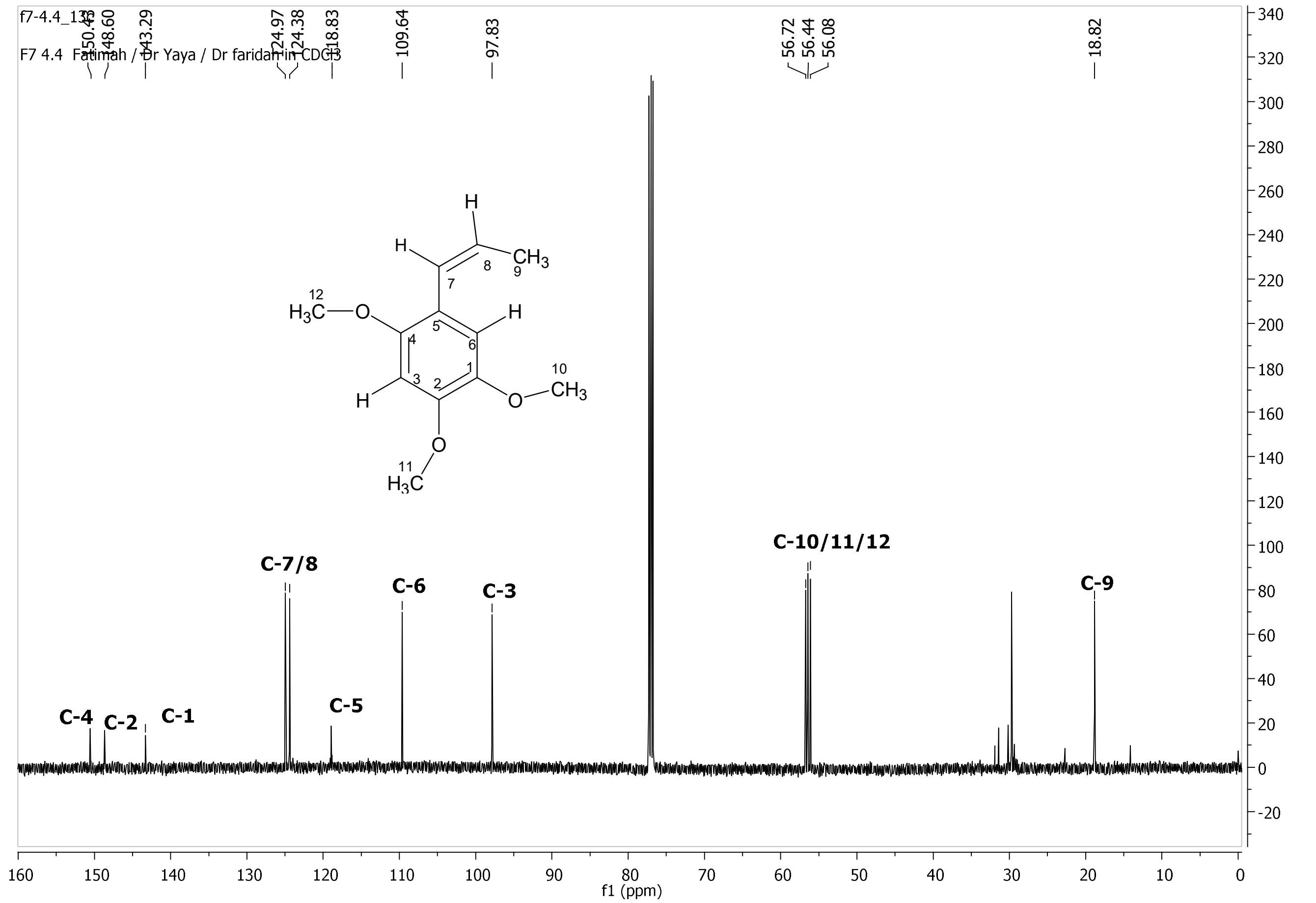

Figure S3. ^{13}C-NMR spectrum of β-asarone (125 MHz, $CDCL_3$).

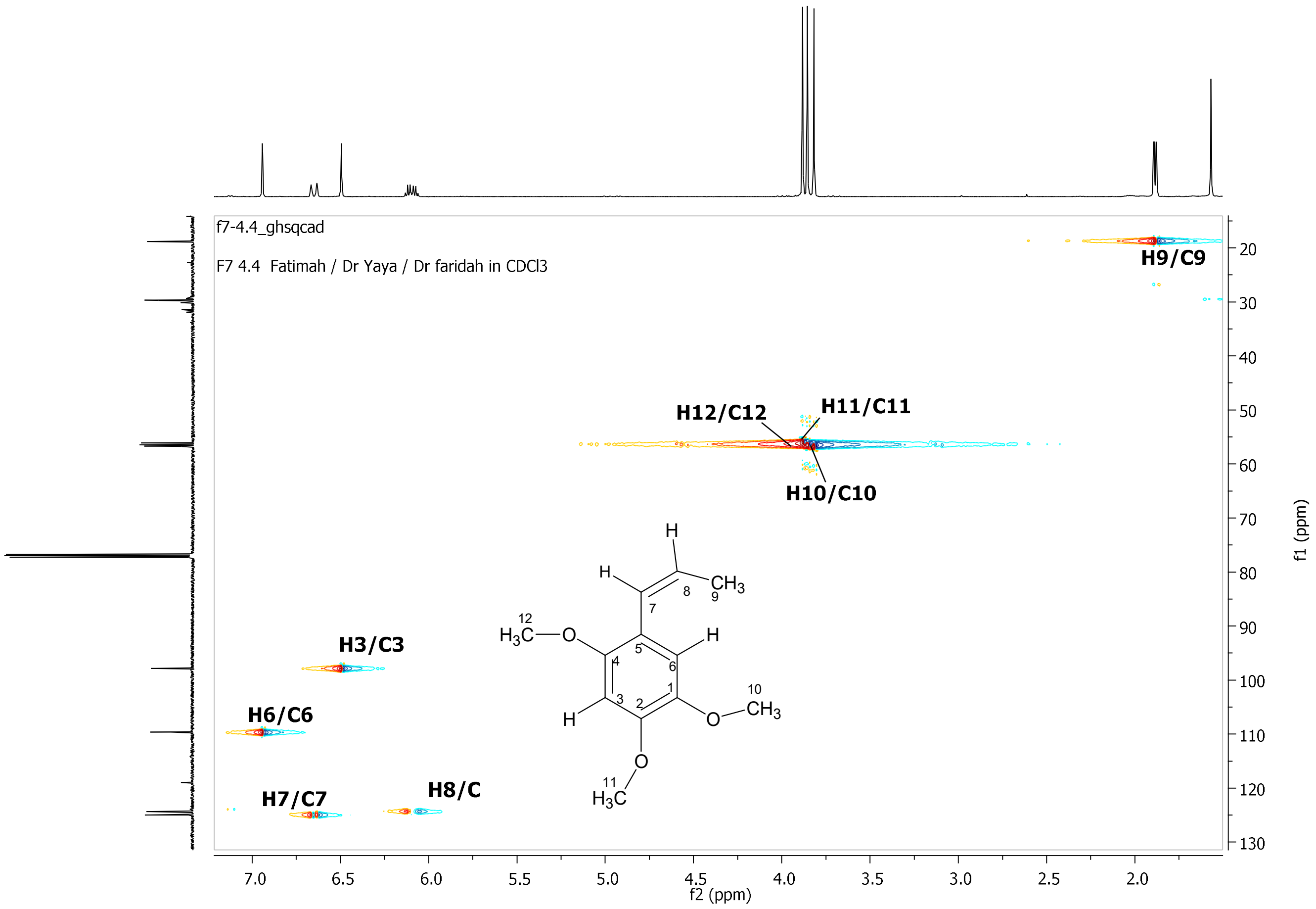

Figure S4. Selected HSQC correlation spectrum of β-asarone.

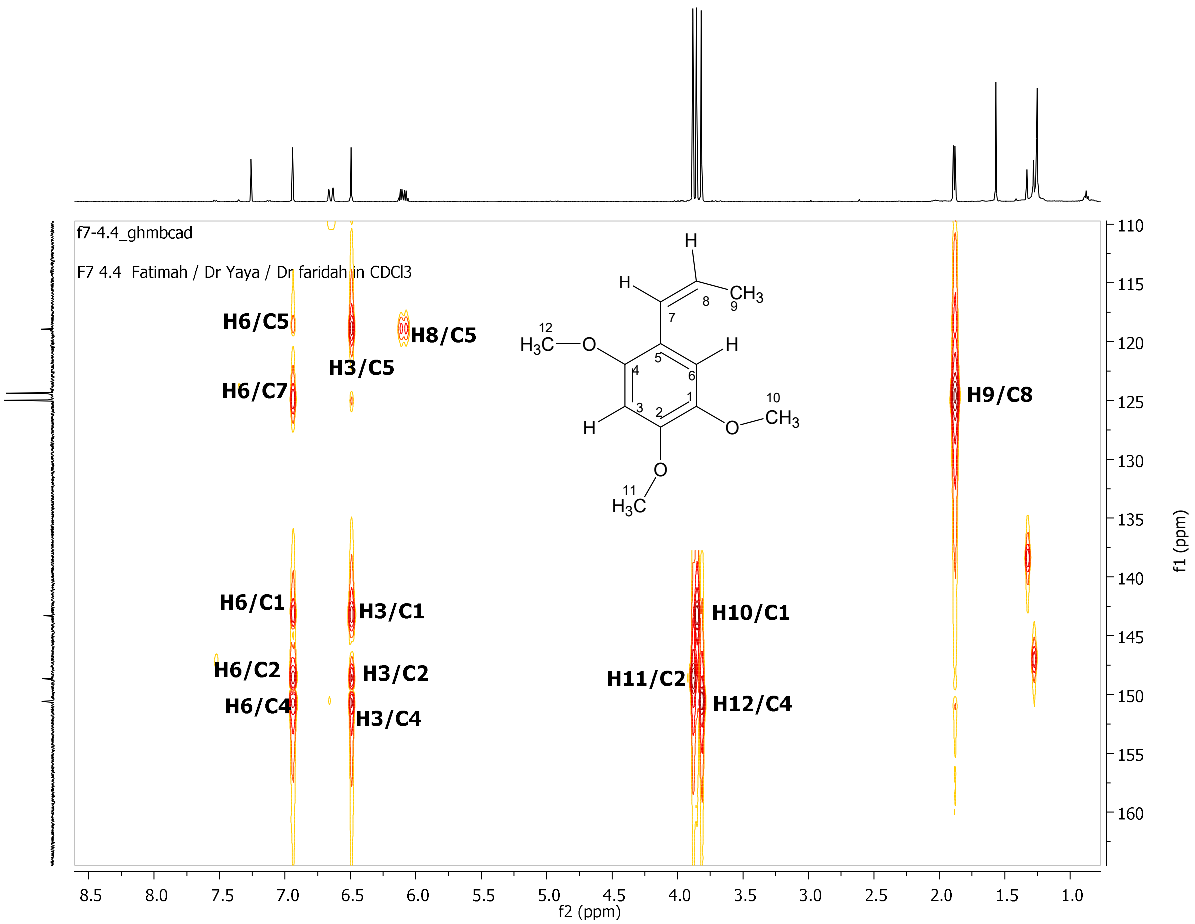

Figure S5. Selected HMBC correlation spectrum of β-asarone.

Table S2. Comparison of ^{1}H-NMR (500 MHz, CDCL$_3$) and ^{13}C-NMR (125 MHz, CDCL$_3$) of compound **(2)** with literature values.

**Position**	$^{\delta}$**H**	$^{\delta}$**C**	**HMBC ^2J**	**HMBC ^3J**	^{1}**H* 500 MHz**	^{13}**C* 125 MHz**
**1**	-	117.5			-	117.3
**2**	-	143.5			-	143.6
**3**	6.49 (1H, s, H-3)	96.1	C-2, C-4		6.50 (1H, s, H-3)	96.0
**4**	-	158.9			-	158.6
**5**	-	156.8			-	155.7
**6**	7.33 (1H, s, H-6)	109.2	C-1, C-5	C-7	7.33(1H, s, H-6)	109.0
**7**	10.32 (1H, s, H-7)	188.3	C-1		10.32(1H, s, H-7)	187.9
**8**	3.97 (3H, s, H-8)	56.46	C-5		3.97 (3H, s, H-8)	56.1
**9**	3.92 (3H, s,H-9)	56.49			3.93 (3H, s, H-9)	56.2
10	3.88 (3H, s, H-10)	56.56	C-2		3.88 (3H, s, H-10)	56.2

*Dung *et al*. (2007)

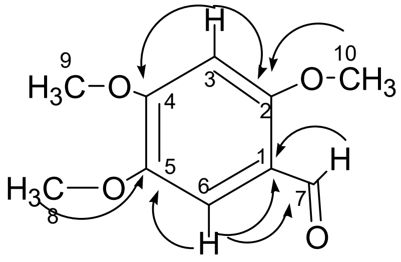

Figure S6. Structure and selected HMBC correlation of asaronaldehyde isolated from *P. cubeba* L.

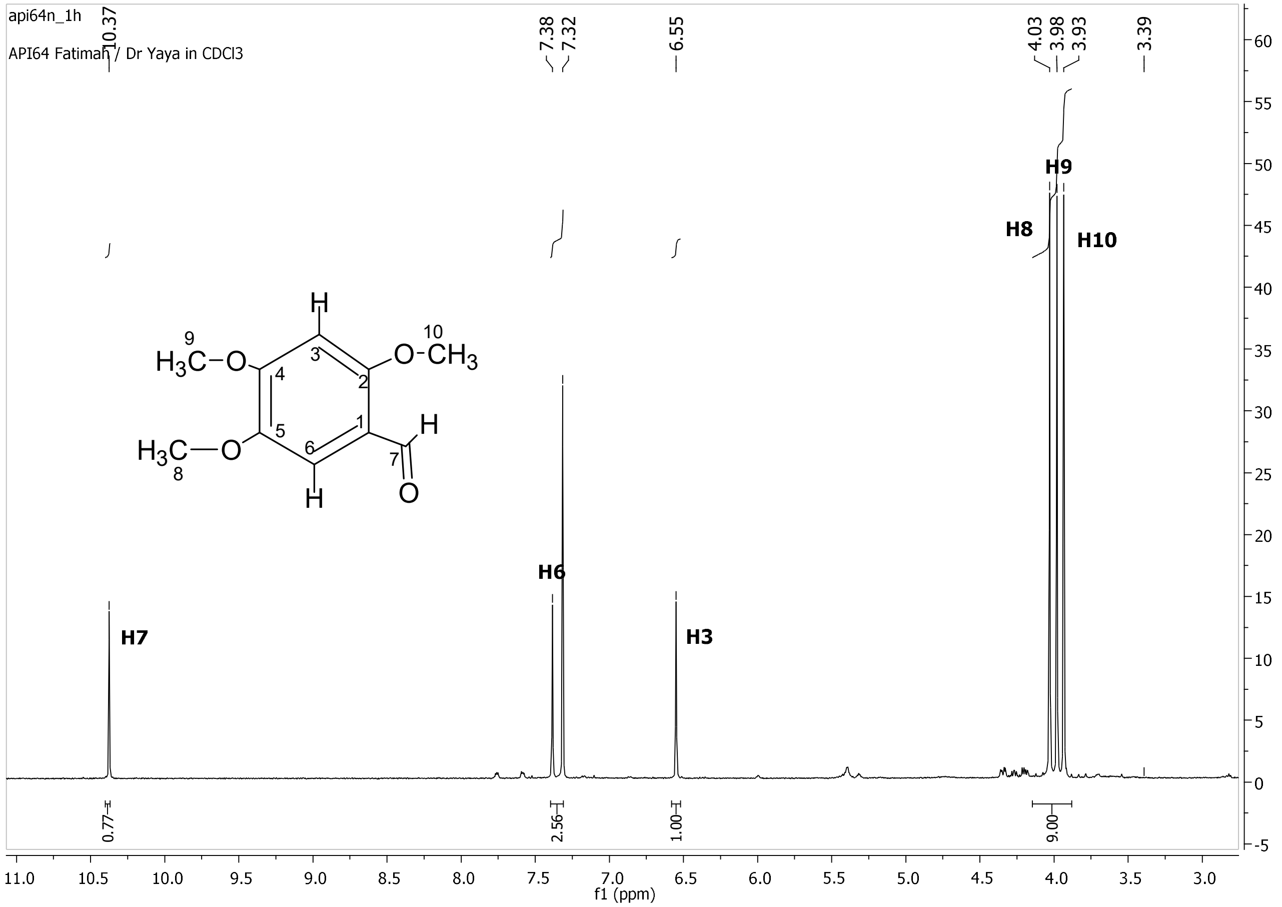

Figure S7. ^{1}H-NMR spectrum of asaronaldehyde (125 MHz, CDCL3).

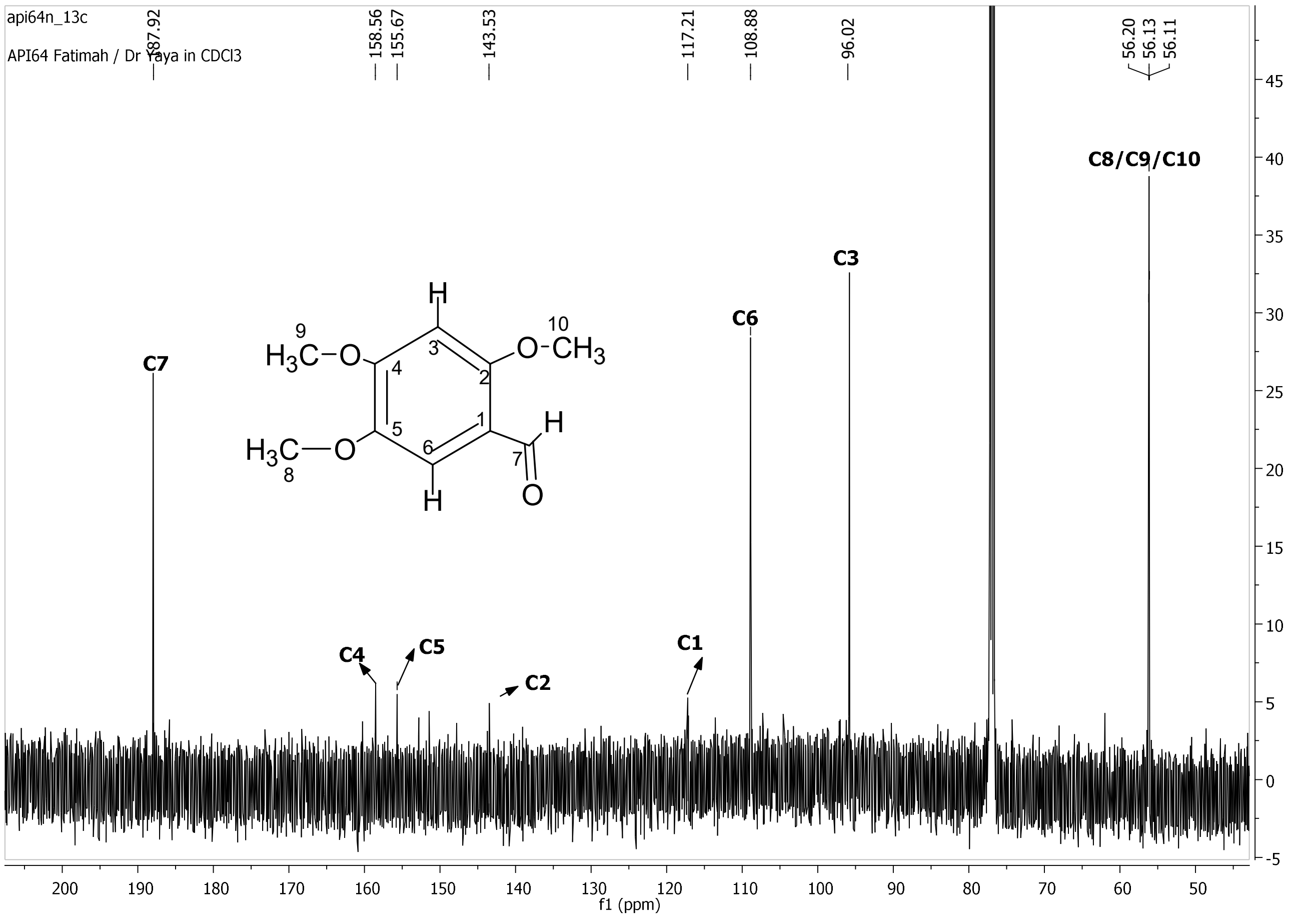

Figure S8. ^{13}C-NMR spectrum of asaronaldehyde (125 MHz, CDCL3).

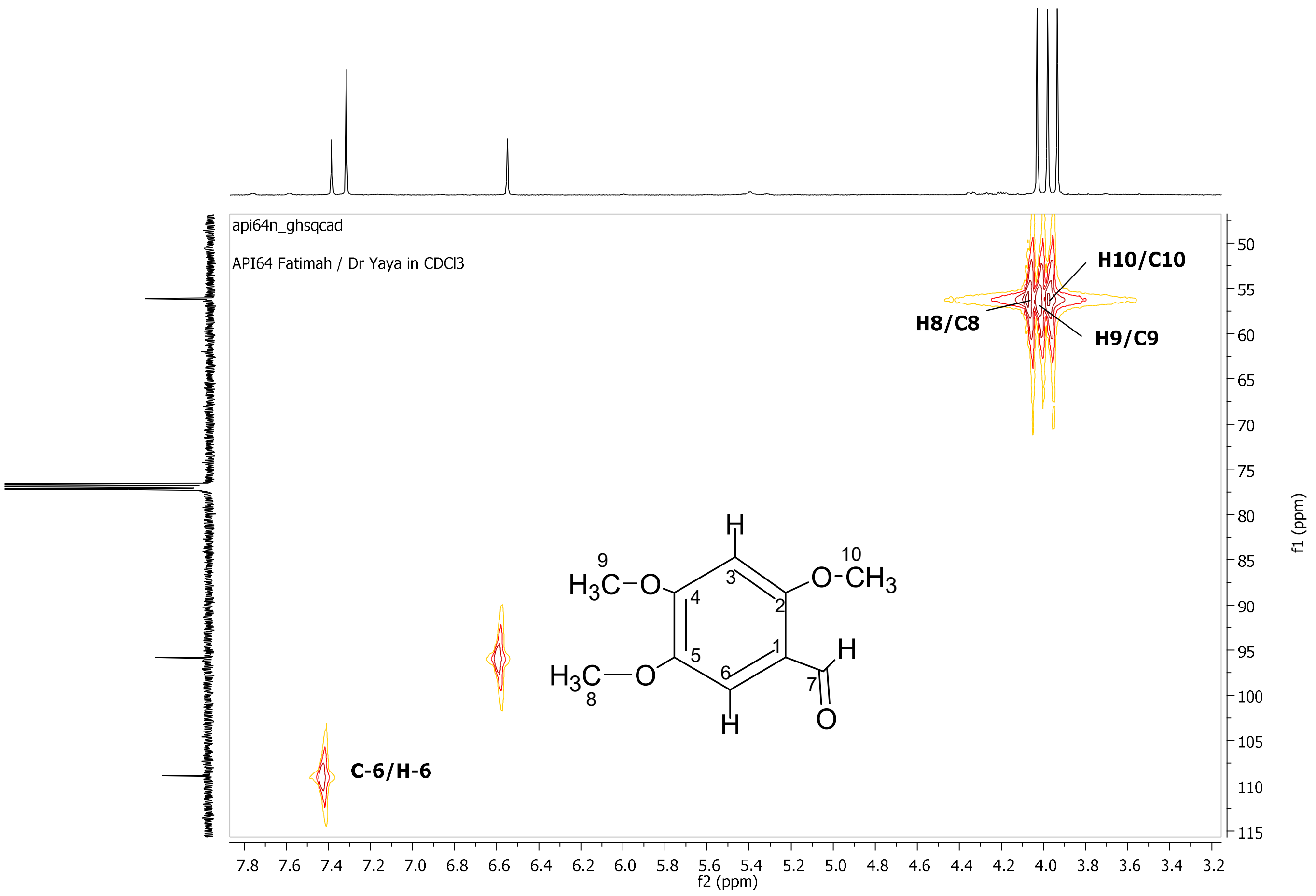

Figure S9. Selected HSQC correlation spectrum of asaronaldehyde.

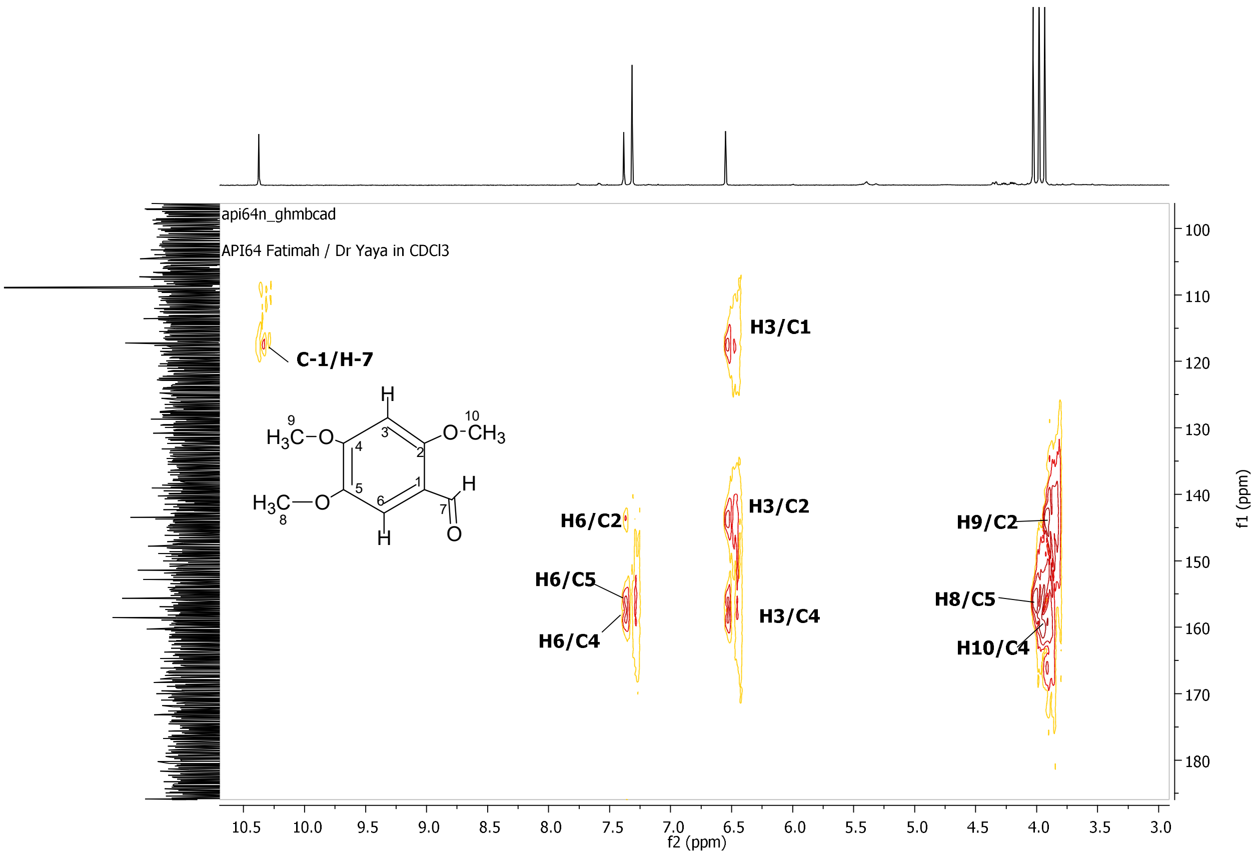

Figure S10. Selected HMBC correlation spectrum of asaronaldehyde.
